# Supplementary material for: Migration of CD8 + TSCM cells into intestine via PPBP–CXCR2 axis increases host stress susceptibility by inhibiting gut microbiome-derived homovanillic acid
Source: Nat Commun. 2025 Nov 19;16:10165. doi: 10.1038/s41467-025-65112-4 (PMC12630981; doi:10.1038/s41467-025-65112-4)
Supplement: Supplementary file 1 — Supplementary Information [file 41467_2025_65112_MOESM1_ESM.pdf]

Supplementary Information

Migration of CD8+ TSCM cells into intestine via PPBP–CXCR2 axis increases host stress susceptibility by inhibiting gut microbiome-derived homovanillic acid

Yuan Zhang,[1†] Minzi Ju,[1†] Suzhen Chen,[2,3†] Wendi Yang,[1†] Yang Cai,[1] Xiaoyu Yu,[1] Gang Chen,[2,3,4] Zhongxia Shen,[2,3,5] Ying Bai,[1] Hui Ren,[1] Yinghui Li,[2,3] Ling Shen,[1] Junxu Li,[6] Peng Shi,[7] Yonggui Yuan,[2,3*] Bing Han,[1,8*]and Honghong Yao[1,9,10*]

[1]Department of Pharmacology, Jiangsu Provincial Key Laboratory of Critical Care Medicine, School of Medicine, Southeast University, Nanjing, Jiangsu, China;

[2]Department of Psychosomatics and Psychiatry, Zhongda Hospital, School of Medicine, Jiangsu Provincial Key Laboratory of Brain Science and Medicine, Southeast University, Nanjing, Jiangsu, China;

[3]Institute of Psychosomatics, School of Medicine, Southeast University, Nanjing, Jiangsu, China;

[4]Department of Psychiatry, the Third People's Hospital of Huai'an, Huai'an, Jiangsu, China;

[5]Department of Psychosomatics, the Third People's Hospital of Huzhou, Huzhou, Zhejiang, China;

[6]Department of Pharmacology and Toxicology, University at Buffalo, Buffalo, NY, USA.

[7]Department of Cardiology of the Second Affiliated Hospital, Zhejiang University School of Medicine, Hangzhou, Zhejiang, China.

[8]Southeast University Affiliated Liangjiang Hospital, Nanjing Pukou People's Hospital, Southeast University, Nanjing 211899, China;

[9]Co-innovation Center of Neuroregeneration, Nantong University, Nantong, Jiangsu, China;

[10]Institute of Life Sciences, Key Laboratory of Developmental Genes and Human Disease, Southeast University, Nanjing, Jiangsu, China.

Supplementary Figures and Legends

Supplementary Fig. 1

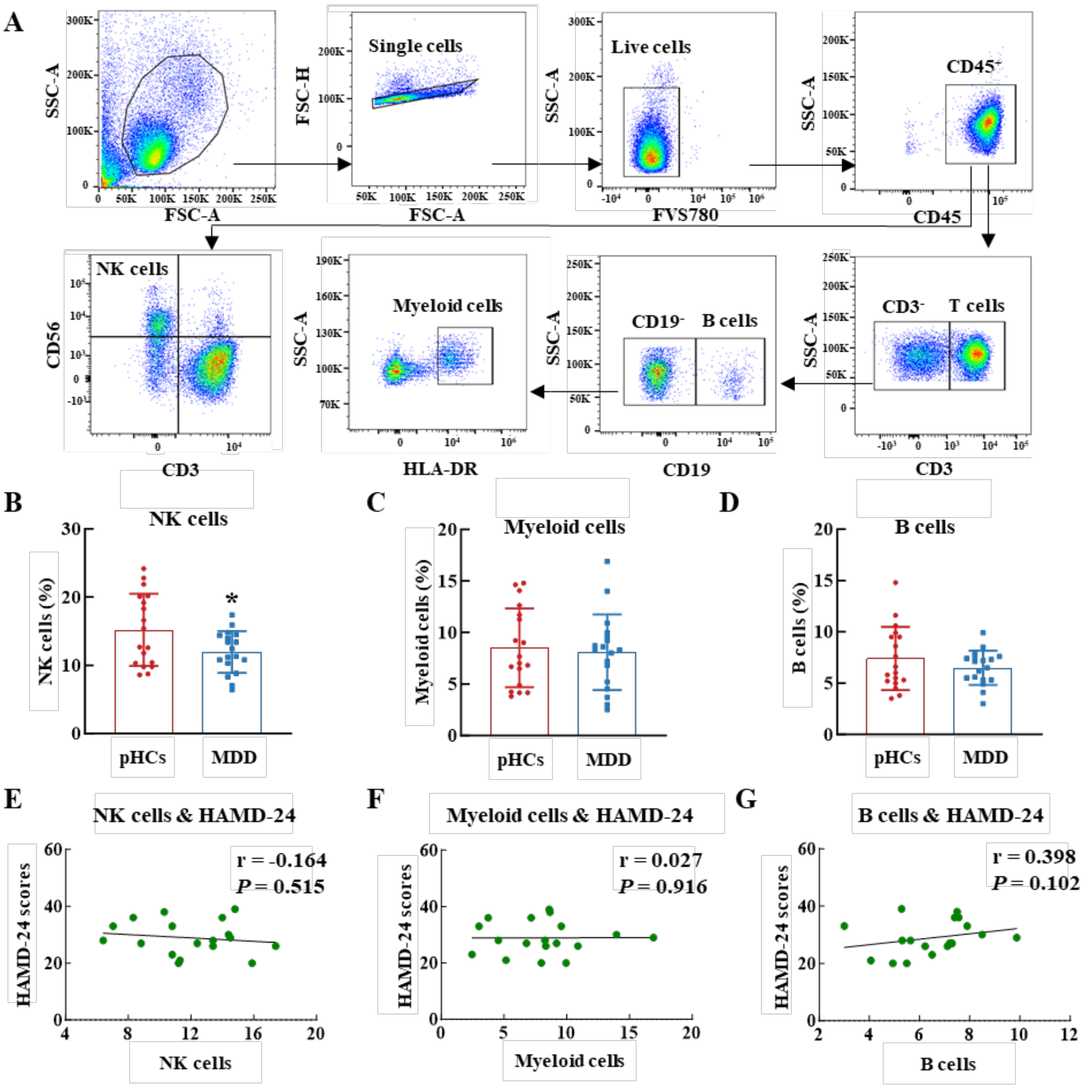

Supplementary Fig. 1 Flow cytometric analysis of human PBMCs in a discovery cohort of pHCs and MDD patients. (A) Flow cytometry gating strategy used to identify immune cell subsets. **(B-D)** Proportions of NK cells (B), myeloid cells (C), and B cells (D) in the pHCs and MDD patients. n = 18/group. *P = 0.031 (B) versus the pHCs group using Student's t test (two-sided). **(E-G)** Correlations between the proportions of NK cells (E), myeloid cells (F), and B cells (G) with HAMD-24 scores in MDD patients using two-sided Pearson's or Spearman's correlation coefficient. n = 18. Data are presented as mean±SD. Source data are provided as a Supplementary Source Data file. pHCs: psychiatrically healthy controls, MDD: major depressive disorder, HAMD-24: the 24-item Hamilton Depression Rating Scale.

Supplementary Fig. 2

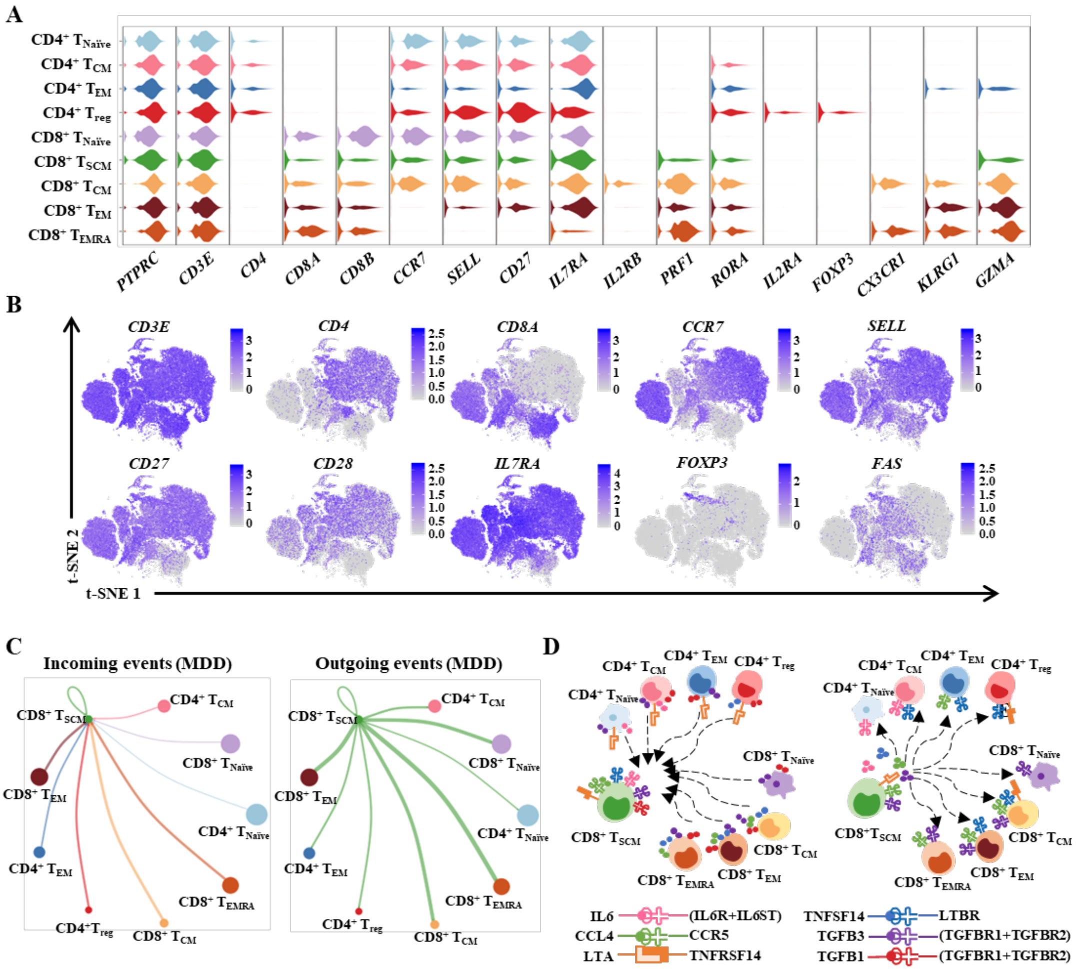

Supplementary Fig. 2 Identification of altered T cell subpopulations in MDD patients. (A) Violin plots showing specific marker genes in nine statistically defined clusters. **(B)** Expression levels of specific marker genes in the T cells were overlaid on the t-SNE representation. **(C)** Circos plots displaying putative ligand–receptor interactions between the CD8$^+$ T_{SCM} subset and other T cell subsets from MDD patients. Interactions are divided into incoming and outgoing events. The brand links pairs of interacting cell types and the corresponding number of events are labeled on the graph. **(D)** Predicted regulatory network centered on CD8$^+$ T_{SCM} cells in MDD patients. MDD: major depressive disorder, T_{SCM} cells: stem cell-like memory T cells, T_{CM} cells: central memory T cells, T_{EM} cells: effector memory T cells, T_{EMRA} cells: terminally differentiated effector memory T cells, $T_{Naïve}$ cells: naïve T cells, T_{reg}: regulatory T cells.

Supplementary Fig. 3

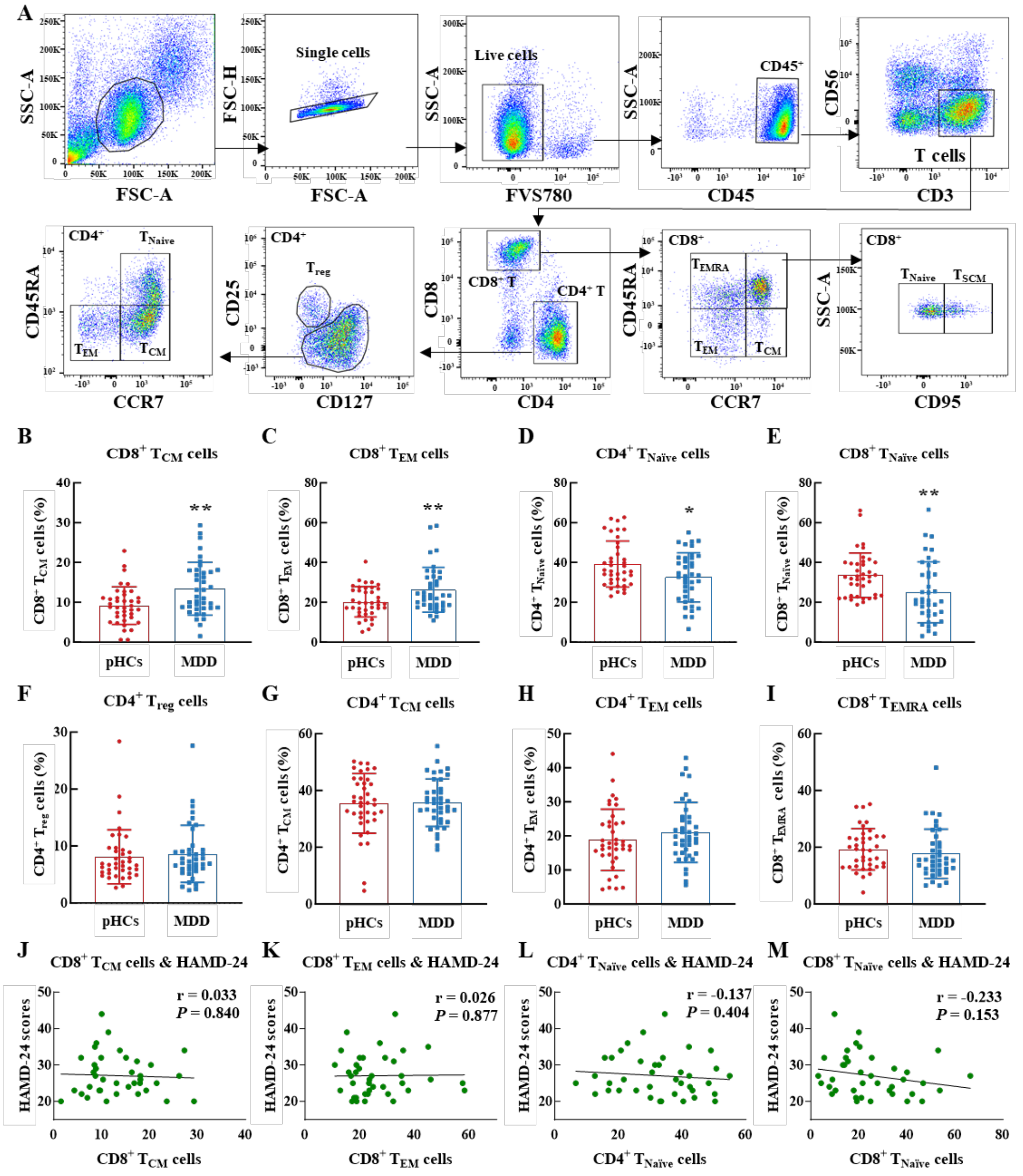

Supplementary Fig. 3 Proportions of T cell subsets in pHCs and MDD patients. (A) Flow cytometry gating strategy was used to identify T cell subsets. **(B-I)** Proportions of CD8$^+$ T_{CM} cells (B), CD8$^+$ T_{EM} cells (C), CD4$^+$ $T_{Naïve}$ cells (D), CD8$^+$ $T_{Naïve}$ cells (E) CD4$^+$ T_{reg} cells (F), CD4$^+$ T_{CM} cells (G), CD4$^+$ T_{EM} cells (H), and CD8$^+$ T_{EMRA} cells (I) in pHCs and MDD patients. *P = 0.0018 (B), **P = 0.0071 (C) and *P = 0.016 (D) versus the pHCs group using Student's t test (two-sided), **P = 0.018 (E) versus the pHCs group using Mann–Whitney U test (two-sided), n = 39/group. **(J-M)** Correlations between CD8$^+$ T_{CM} (J), CD8$^+$ T_{EM} (K), cell CD4$^+$ $T_{Naïve}$ (L), and CD8$^+$ $T_{Naïve}$ (M) cell proportions and HAMD-24 scores in MDD patients using two-sided Pearson's or Spearman's correlation coefficient. n = 39. Data are presented as mean±SD. Source data are provided as a Supplementary Source Data file. pHCs: psychiatrically healthy controls, MDD: major depressive disorder, $T_{Naïve}$ cells: naïve T cells, T_{CM} cells: central memory T cells, T_{EM} cells: effector memory T cells, T_{reg}: regulatory T cells, T_{EMRA} cells:

terminally differentiated effector memory T cells, T_{SCM} cells: stem cell-like memory T cells, HAMD-24: the 24-item Hamilton Depression Rating Scale.

Supplementary Fig. 4

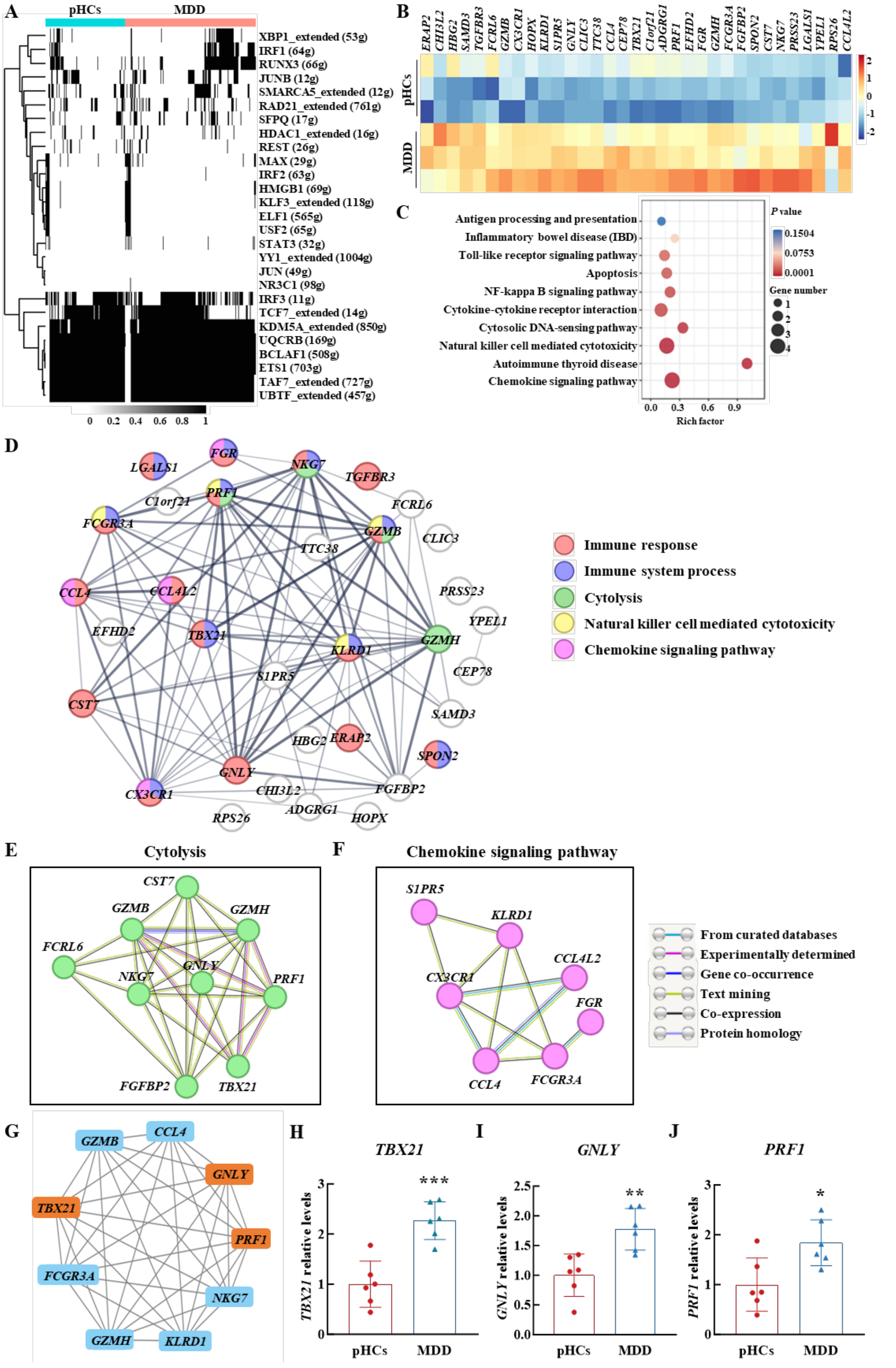

Supplementary Fig. 4 PPI network analysis of CD8$^+$ T$_{SCM}$ cells in MDD patients. (A) Heatmap of SCENIC binary regulon activities of CD8+ TSCM cells. Three TF regulatory networks with high activities in CD8+ TSCM cells in MDD patients were highlighted. **(B)** Heatmap showing the DEGs in CD8$^+$ T$_{SCM}$ cells from MDD patients

and pHCs. **(C)** KEGG analysis showing the major signaling pathways involving the DEGs. **(D)** STRING network results for the DEGs; nodes corresponded to a set of biological processes and pathways highlighted. **(E)** Subset of genes involved in cytolysis. **(F)** Subset of genes involved in chemokine signaling pathway. **(G)** Visualization of the 3 hub genes identified by the combination of CytoHubba and MCODE in Cytoscape. **(H-J)** Relative expression of *TBX21* (G), *GNLY* (H) and *PRF1* (I) in the sorted $CD8^+$ T_{SCM} cells as determined by qPCR. $n = 6$ samples (the $CD8^+$ T_{SCM} cells isolated from six pHCs or MDD patients were pooled for one sample). $^{***}P = 0.0004$ (G), $^{**}P = 0.0036$ (H) and $^{*}P = 0.0152$ (I) versus the pHCs group using Student's *t* test (two-sided). Data are presented as mean±SD. Source data are provided as a Supplementary Source Data file. pHCs: psychiatrically healthy controls, MDD: major depressive disorder, $CD8^+$ T_{SCM} cells: stem cell-like memory $CD8^+$ T cells.

Supplementary Fig. 5

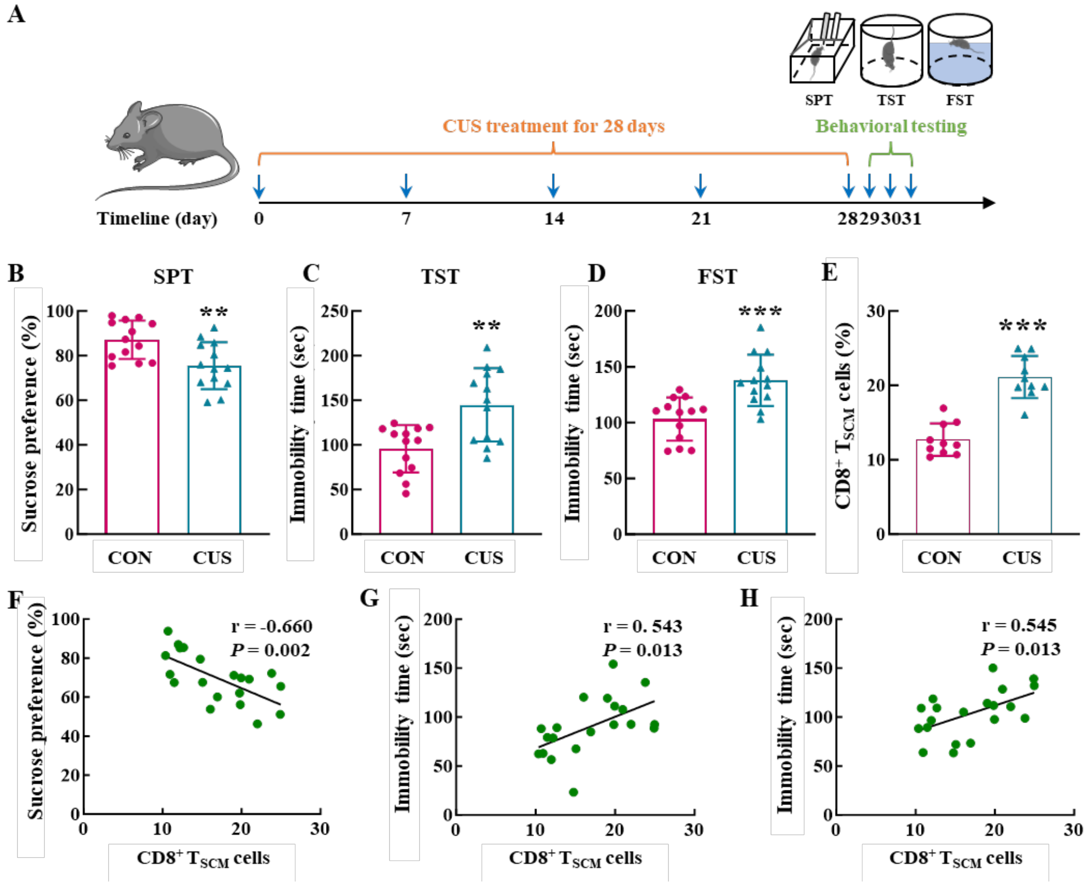

Supplementary Fig. 5 CUS protocol was applied to induce depressive-like behaviors in mice. (A) Schematic of the experimental procedure and behavioral studies. Components of this figure were created using Servier Medical Art templates, which are licensed under a Creative Commons Attribution 3.0 Unported License; https://smart.servier.com. **(B-D)** The depressive-like behaviors of CUS mice were evaluated by SPT (B), TST (C), and FST (D). n = 13/group. **(E)** Proportions of $CD8^+$ T_{SCM} cells were increased in CUS group. n = 10/group. $^{**}P = 0.005$ (B), $^{**}P = 0.0013$ (C), $^{***}P = 0.0003$ (D) and $^{***}P < 0.0001$ (E) versus the control group using Student's t test (two-sided). **(F-H)** The correlations of $CD8^+$ T_{SCM} cell proportions with the sucrose uptake in SPT (F) and the immobility time in both TST (G) and FST (H) using two-sided Pearson's or Spearman's correlation coefficient. n = 20. Data are presented as mean±SD. Source data are provided as a Supplementary Source Data file. CON: control, CUS: chronic unpredictable stress, SPT: sucrose preference test, TST: tail suspension test, FST: forced swim test, $CD8^+$ T_{SCM} cells: stem cell-like memory $CD8^+$ T cells.

Supplementary Fig. 6

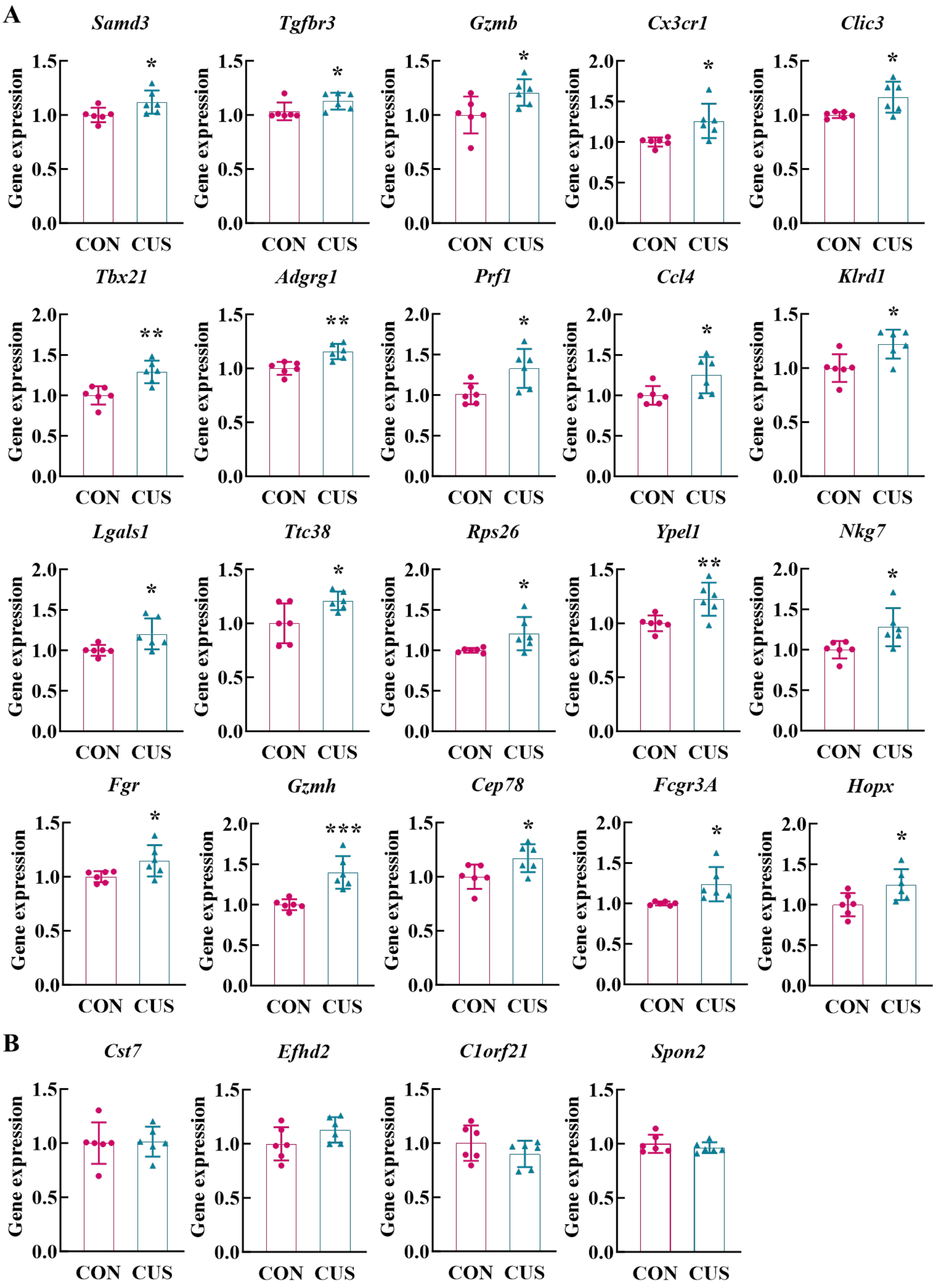

Supplementary Fig. 6 The expression of DEGs in CD8⁺ T$_{SCM}$ cells from CON or CUS mice. (A) Twenty DEGs were higher expression in CD8⁺ T$_{SCM}$ cells from CUS mice compared with in CD8⁺ T$_{SCM}$ cells from CON group. **(B)** There was on significant difference expression of four DEGs in CD8⁺ T$_{SCM}$ cells between CON and CUS mice. *n* = 6/group. **P* = 0.0437 (*Samd3*), **P* = 0.0411 (*Tgfbr3*), **P* = 0.0356 (*Gzmb*), **P* =

0.0158 (*Cx3cr1*), *P = 0.0197 (*Clic3*), **P = 0.0027 (*Tbx21*), **P = 0.0021 (*Adgrg1*), *P = 0.0181 (*Prf1*), *P = 0.0356 (*Ccl4*), *P = 0.015 (*Klrd1*), *P = 0.0338 (*Lgals1*), *P = 0.0303 (*Ttc38*), *P = 0.0349 (*Rps26*), **P = 0.009 (*Ypel1*), *P = 0.0255 (*Nkg7*), *P = 0.0405 (*Fgr*), ***P = 0.001 (*Gzmh*), *P = 0.0339 (*Cep78*), *P = 0.0214 (*Fcgr3A*), and *P = 0.0307 (*Hopx*) versus the control group using Student's *t* test (two-sided) or the Mann–Whitney *U* test (two-sided). Data are presented as mean±SD. Source data are provided as a Supplementary Source Data file. CON: control, CUS: chronic unpredictable stress.

Supplementary Fig. 7

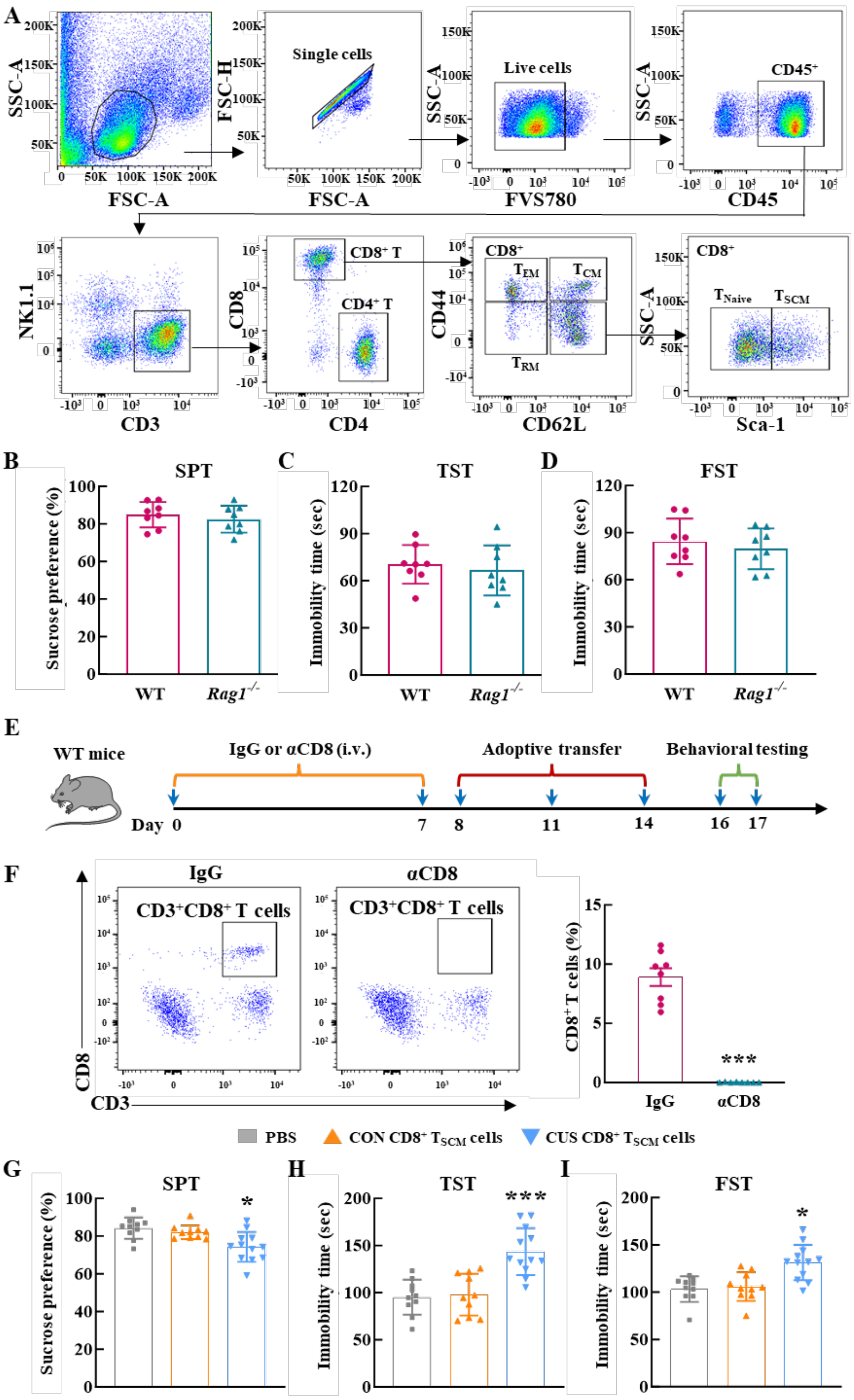

Supplementary Fig. 7 CD8$^+$ T cells depleted in Wild-type (WT) C57BL/6J mice by αCD8. (A) Gating strategy for sorting CD8$^+$ T_{SCM} cells by flow cytometry. **(B-D)** The depressive-like behaviors of WT and *Rag1*$^{-/-}$ mice were evaluated by the SPT (B), TST (C), and FST (D). n = 8/group. **(E)** Schematic showing the experimental design for CD8$^+$ T cells depletion and CD8$^+$ T_{SCM} cell adoptive transfer. Components of this figure

were created using Servier Medical Art templates, which are licensed under a Creative Commons Attribution 3.0 Unported License; https://smart.servier.com. **(F)** Gating strategy for identifying $CD3^+CD8^+$ T cell populations by flow cytometry. Proportions of $CD8^+$ T cells belonging to each sample. ****P* = 0.0002 versus the IgG group using Mann-Whitney *U* test (two-sided), *n* = 8/group. **(G-I)** The depressive-like behaviors of recipient WT mice were evaluated by the SPT (G), TST (H) and FST (I). PBS (*n* = 10), CON $CD8^+$ T_{SCM} cells (*n* = 10), CUS $CD8^+$ T_{SCM} cells (*n* = 12). ***P*** = 0.0122 (G) and *****P*** < 0.0001 (H) versus the control $CD8^+$ T_{SCM} cells group using one-way ANOVA followed by two-sided Holm-Sidak post hoc multiple comparison test. ***P*** = 0.0105 (I) versus the control $CD8^+$ T_{SCM} cells group using Kruskal-Wallis test followed by two-sided Dunn's post hoc multiple comparison test. Data are presented as mean±SD. Source data are provided as a Supplementary Source Data file. CON: control, CUS: chronic unpredictable stress, SPT: sucrose preference test, TST: tail suspension test, FST: forced swim test, T_{CM} cells: central memory T cells, T_{EM} cells: effector memory T cells, T_{RM} cells: Tissue-resident memory T cells, $T_{Naïve}$ cells: naïve T cells, T_{SCM} cells: stem cell-like memory T cells.

Supplementary Fig. 8

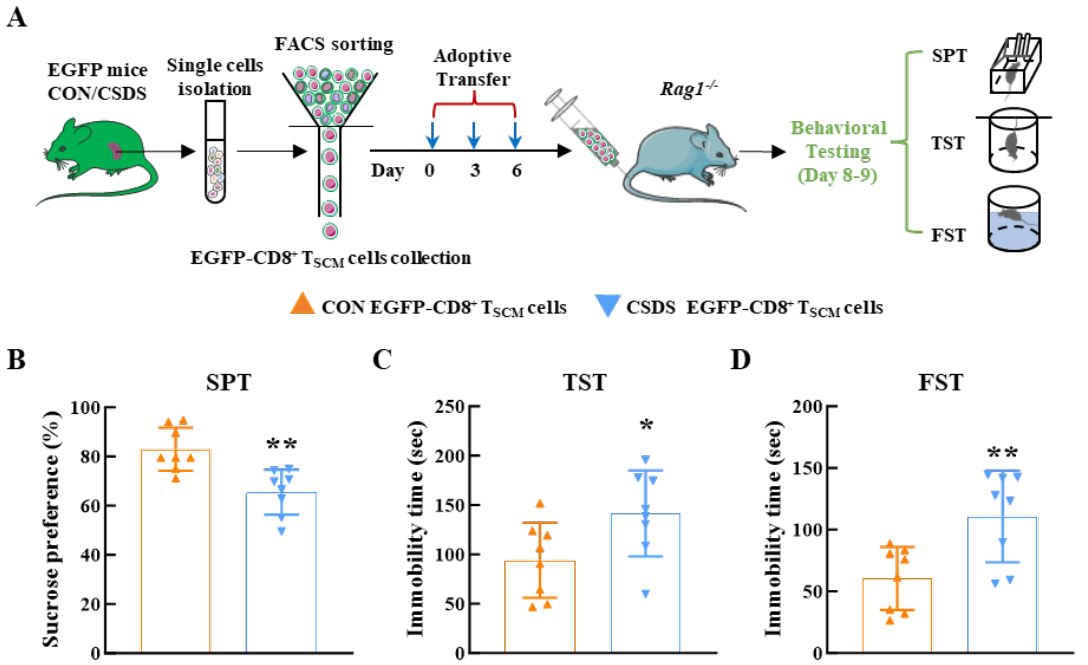

Supplementary Fig. 8 The depressive-like behaviors of recipient *Rag1*$^{-/-}$ mice. (A) Schematic showing the experimental design for EGFP labeled-CD8^{+} T$_{SCM}$ cell adoptive transfer. Components of this figure were created using Servier Medical Art templates, which are licensed under a Creative Commons Attribution 3.0 Unported License; https://smart.servier.com. **(B-D)** The depressive-like behaviors of recipient *Rag1*$^{-/-}$ mice were evaluated by the SPT (B), TST (C) and FST (D). n = 8/group. $^{**}P$ = 0.0016 (B), $^{*}P$ = 0.0359 (C) and $^{**}P$ = 0.0071 (D) versus the CON EGFP-CD8^{+} T$_{SCM}$ group using Student's t test (two-sided). Data are presented as mean±SD. Source data are provided as a Supplementary Source Data file. CON: control, CSDS: chronic social defeat stress, EGFP: enhanced green fluorescent protein, SPT: sucrose preference test, TST: tail suspension test, FST: forced swim test, CD8^{+} T$_{SCM}$ cells: stem cell-like memory CD8^{+} T cells.

Supplementary Fig. 9

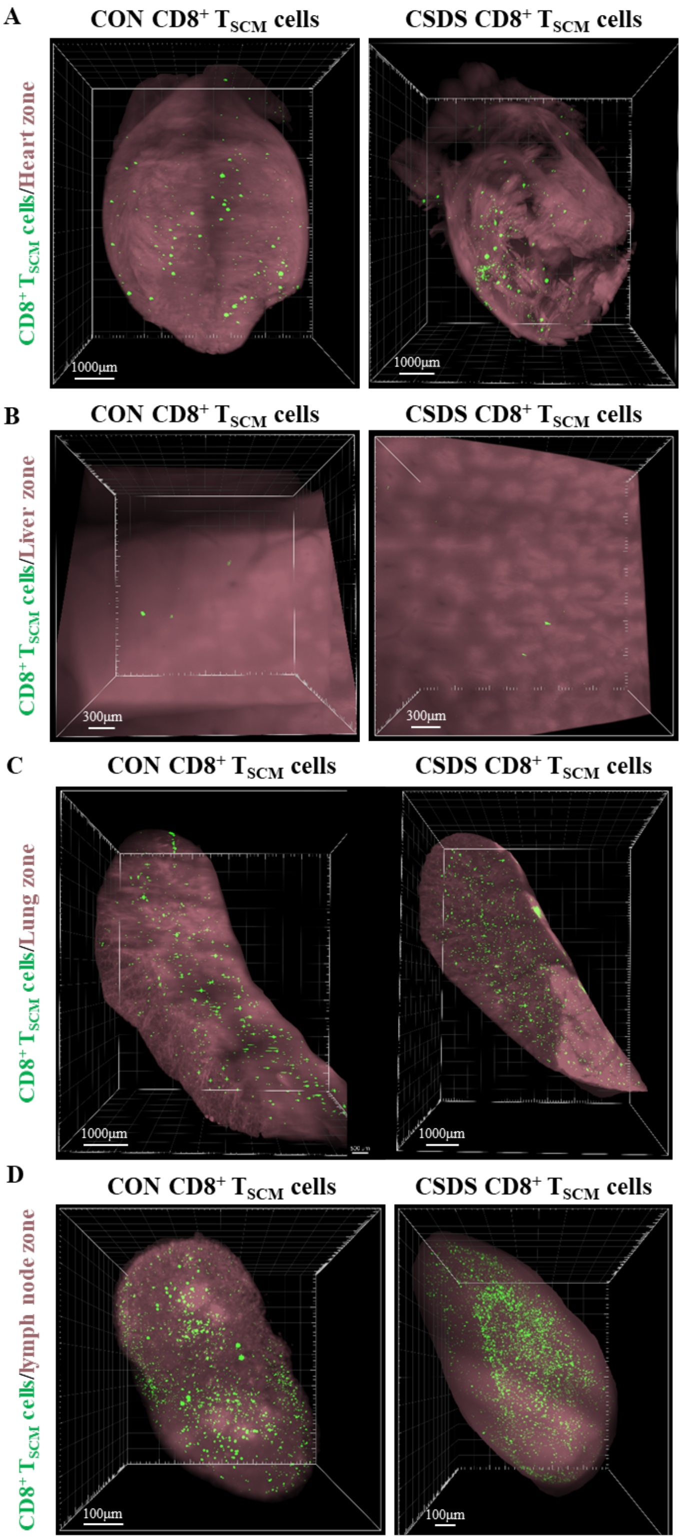

Supplementary Fig. 9 Image of EGFP labeled-CD8^{+} T$_{SCM}$ cells in lung, heart and liver of *Rag1*$^{-/-}$ mice. (A-D) The EGFP labeled-CD8^{+} T$_{SCM}$ cells in heart (A), liver (B), lung (C) and lymph node (D) were detected by vDISCO, a pressure-driven, nanobody-based whole-body immunolabeling technology and imaged by light sheet microscopy. CON: control, CSDS: chronic social defeat stress, CD8^{+} T$_{SCM}$ cells: stem cell-like memory CD8^{+} T cells.

Supplementary Fig. 10

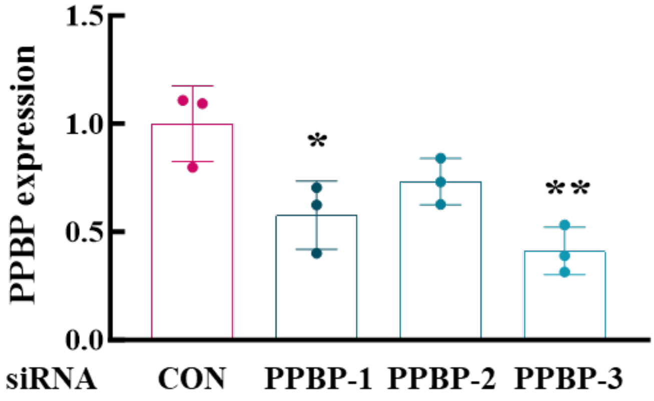

Supplementary Fig. 10 The expression of PPBP in 293T cells treated with siPPBP lentivirus. The cells were treated with three different siPPBP lentivirus targeting different sequence (PPBP-1, PPBP-2, PPBP-3). The PPBP expression were decreased by the siPPBP-1 and siPPBP-3 lentivirus. ******P* = 0.03 and *******P* = 0.0053 versus the siRNA control group using one-way ANOVA followed by two-sided Holm-Sidak post hoc multiple comparison test, *n* = 3/group. Data are presented as mean±SD. Source data are provided as a Supplementary Source Data file. CON, control group.

Supplementary Fig. 11

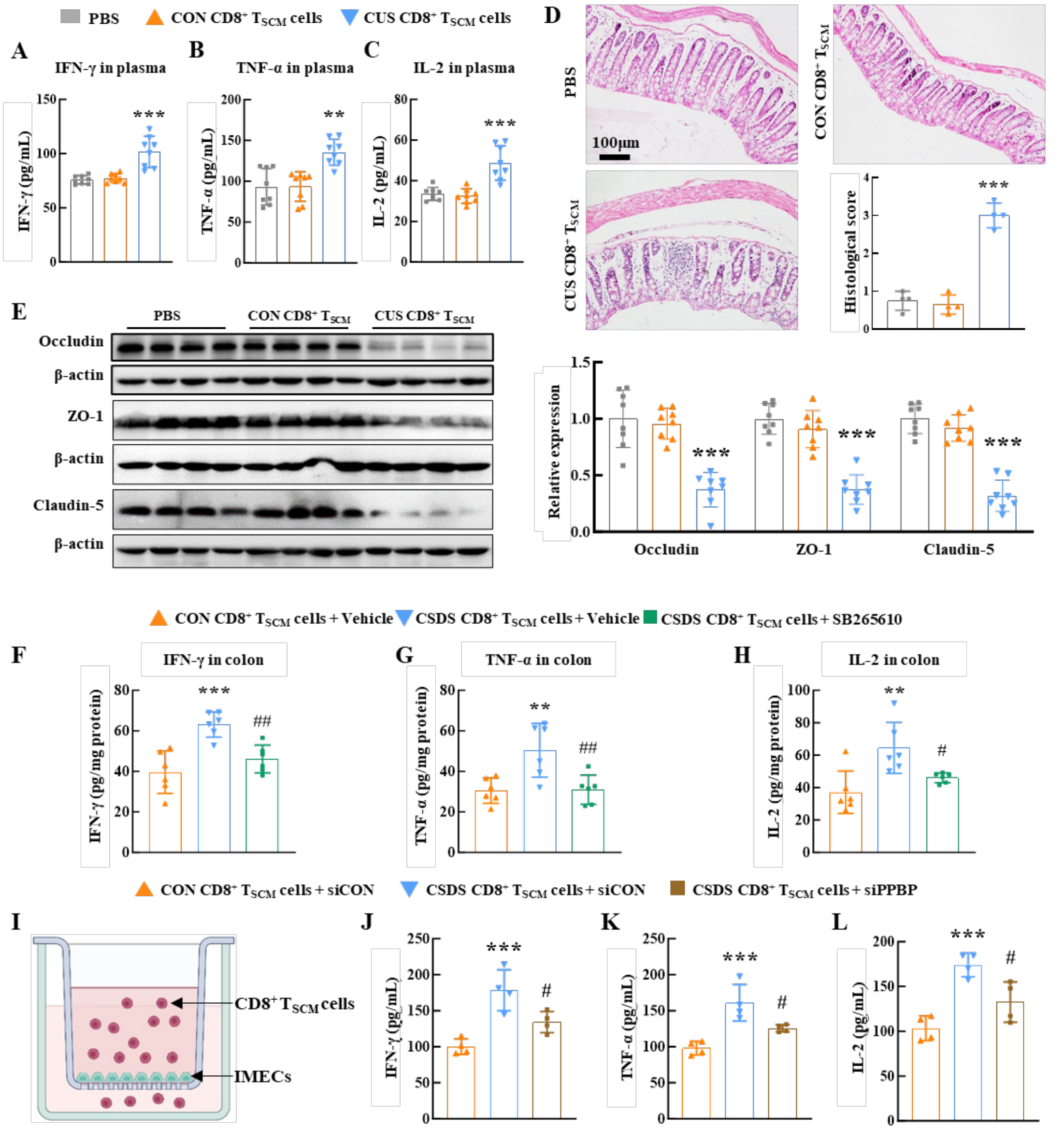

Supplementary Fig. 11 The pathological CD8$^+$ T$_{SCM}$ cells induced the inflammation of intestine. (A-C) The level of IFN-γ (A), TNF-α (B) and IL-2 (C) measured by ELISA in the plasma. ****P* < 0.0001 (A) and ****P* < 0.0001 (C) versus the control CD8$^+$ T$_{SCM}$ cells group using one-way ANOVA followed by two-sided Holm-Sidak post hoc multiple comparison test, ***P* = 0.0023 (B) versus the control CD8+ TSCM cells group using Kruskal-Wallis test followed by two-sided Dunn's post hoc multiple comparison test, *n* = 8/group. **(D)** Representative H&E-stained sections and histologic scores in the colon. ****P* < 0.0001 versus the control CD8$^+$ T$_{SCM}$ cells group using one-way ANOVA followed by two-sided Holm-Sidak post hoc multiple comparison test, *n* = 4/group. **(E)** Representative western blots showing the levels of TJPs in the colon. ****P* < 0.0001 (Occludin), ****P* < 0.0001 (ZO-1) and ****P* < 0.0001 (Claudin-5) versus the control CD8$^+$ T$_{SCM}$ cells group using one-way ANOVA followed by two-sided Holm-Sidak post hoc multiple comparison test, *n* = 8/group. **(F-H)** The level of IFN-γ (F), TNF-α (G) and IL-2 (H) measured by ELISA in colon. ****P* = 0.0004 (F), ***P* = 0.0071 (G) and ***P* = 0.0037 (H) versus the control CD8$^+$ T$_{SCM}$ cells group, $^{\#\#}$*P* = 0.0048 (F), $^{\#\#}$*P* = 0.0071 (G) and $^{\#}$*P* = 0.0348 (H) versus the CSDS CD8$^+$

T_{SCM} cells group using one-way ANOVA followed by two-sided Holm-Sidak post hoc multiple comparison test, *n* = 6/group. **(G)** Schematic depicting the experimental setup of transwell. **(I)** Schematic depicting the experimental setup of transwell. Created in BioRender. Yao, H. (2025) https://BioRender.com/iqfc57z. **(J-L)** The level of IFN-γ (J), TNF-α (K) and IL-2 (L) measured by ELISA in the lower chamber of transwell system. ****P* = 0.0009 (J), ****P* = 0.001 (K) and ****P* = 0.0007 (L) versus the control $CD8^+$ T_{SCM} cells group, $^{\#}P$ = 0.0209 (J), $^{\#}P$ = 0.0229 (K) and $^{\#}P$ = 0.0147 (L) versus the CSDS $CD8^+$ T_{SCM} cells group using one-way ANOVA followed by two-sided Holm-Sidak post hoc multiple comparison test, *n* = 4/group. Data are presented as mean±SD. Source data are provided as a Supplementary Source Data file. CON: control, CUS: chronic unpredictable stress, CSDS: chronic social defeat stress, $CD8^+$ T_{SCM} cells: stem cell-like memory $CD8^+$ T cells.

Supplementary Fig. 12

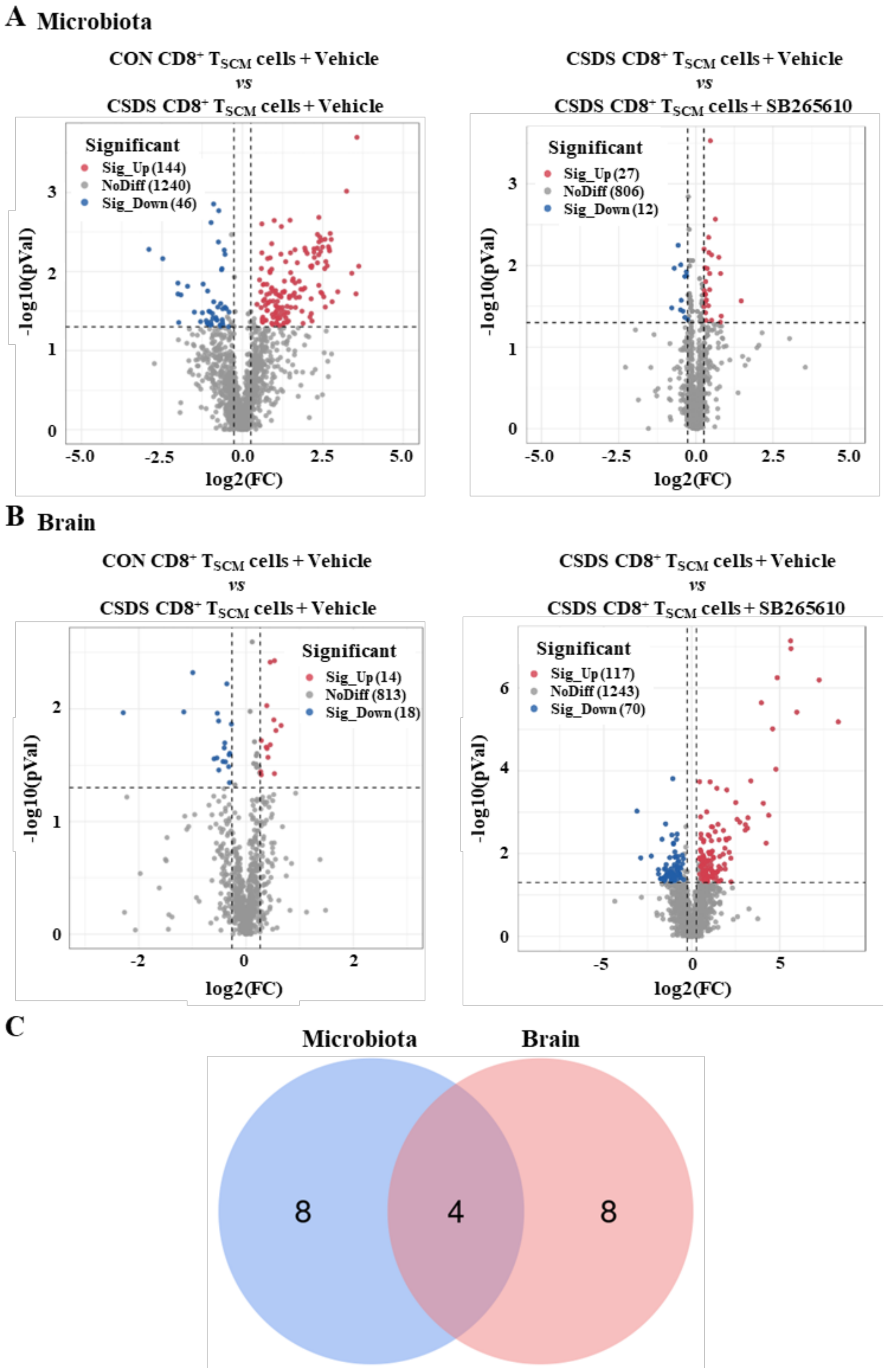

Supplementary Fig. 12 Metabolomics analysis in microbiota and brain. (A-B) The volcano plot showed the differential metabolites in microbiota (A) and brain tissue (B) between group CON $CD8^+$ T_{SCM} cells+Vehicle and CSDS $CD8^+$ T_{SCM} cells+Vehicle/group CSDS $CD8^+$ T_{SCM} cells+Vehicle and CSDS $CD8^+$ T_{SCM} cells+SB265610. **(C)** The Venn Diagram showed four KEGG pathways were both in microbiota and brain tissue. Data are presented as mean±SD. Source data are provided as a Supplementary Source Data file. CON: control, CSDS: chronic social defeat stress, $CD8^+$ T_{SCM} cells: stem cell-like memory $CD8^+$ T cells.

Supplementary Fig. 13

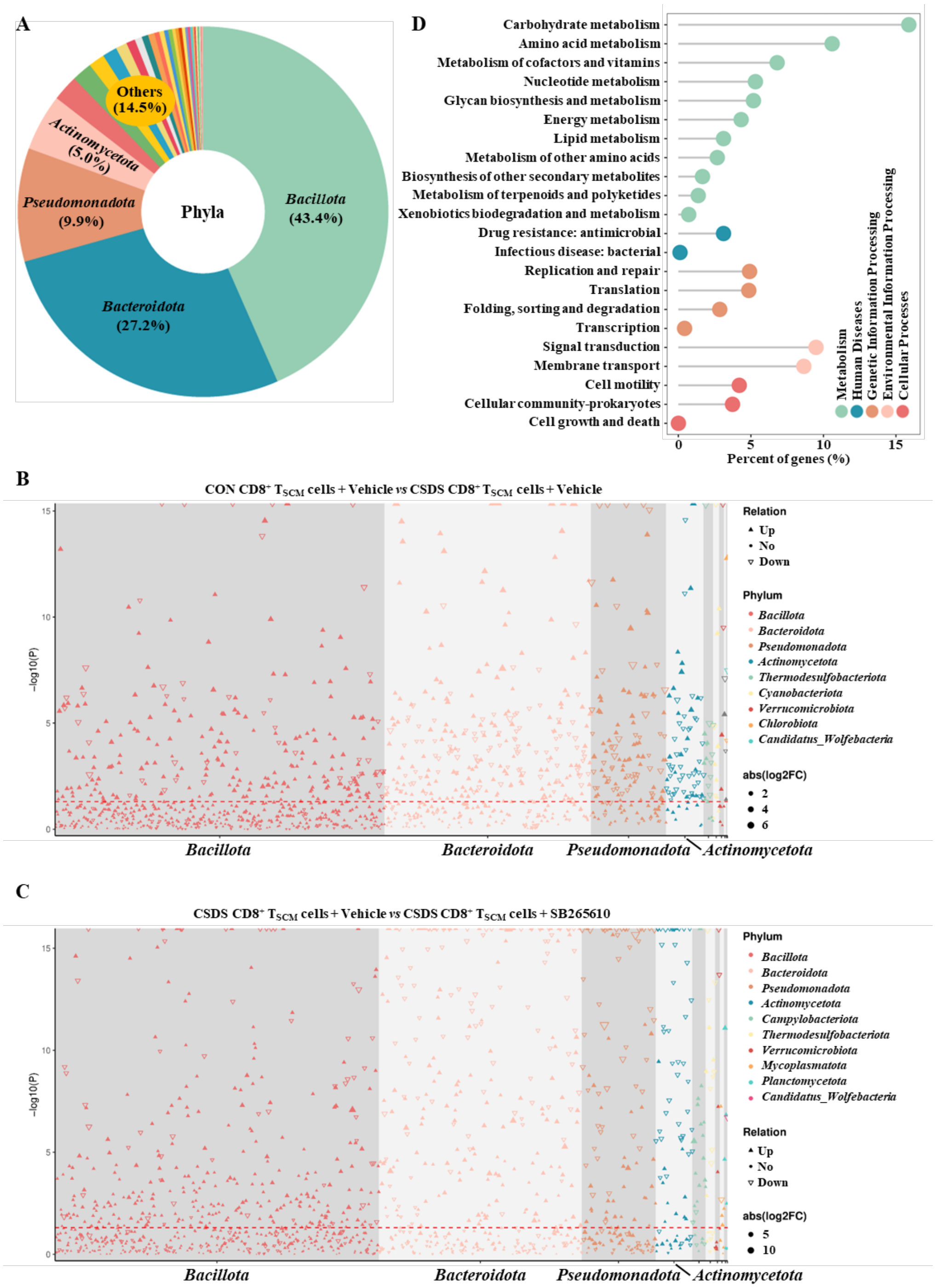

Supplementary Fig. 13 Metagenomic sequencing analysis in microbiota. (A) The identified differential microbiota belonged to four phyla. **(B-C)** The Manhattan plot showed the differential microbiota between CON CD8$^+$ T$_{SCM}$ cells+Vehicle group and CSDS CD8$^+$ T$_{SCM}$ cells+Vehicle group. **(B)** The Manhattan plot showed the differential microbiota between CSDS CD8$^+$ T$_{SCM}$ cells+Vehicle group and CSDS CD8$^+$ T$_{SCM}$ cells+SB265610 group. **(D)** The KEGG pathway enrichment analysis in the differential bacteria. Data are presented as mean±SD. Source data are provided as a Supplementary Source Data file. CON: control, CSDS: chronic social defeat stress, CD8$^+$ T$_{SCM}$ cells: stem cell-like memory CD8$^+$ T cells.

Supplementary Fig. 14

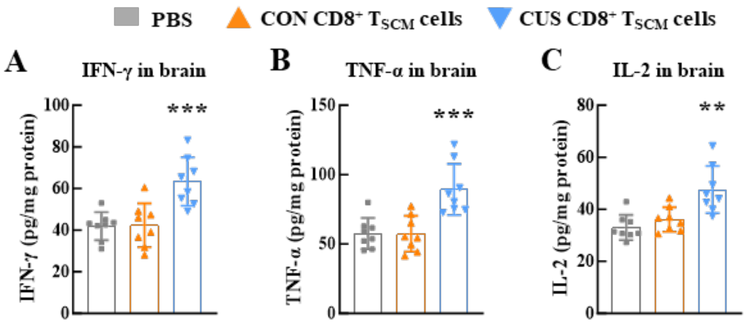

Supplementary Fig. 14 The pathological CD8$^+$ T$_{SCM}$ cells induced neuroinflammation. (A-C) The level of IFN-γ (A), TNF-α (B) and IL-2 (C) measured by ELISA in the brain tissue. ***P = 0.0008 (A), ***P = 0.0007 (B) and **P = 0.004 (C) versus the control CD8$^+$ T$_{SCM}$ cells group using one-way ANOVA followed by two-sided Holm-Sidak post hoc multiple comparison test, n = 8/group. Data are presented as mean±SD. Source data are provided as a Supplementary Source Data file. CON: control, CUS: chronic unpredictable stress, CD8$^+$ T$_{SCM}$ cells: stem cell-like memory CD8$^+$ T cells.

Supplementary Fig. 15

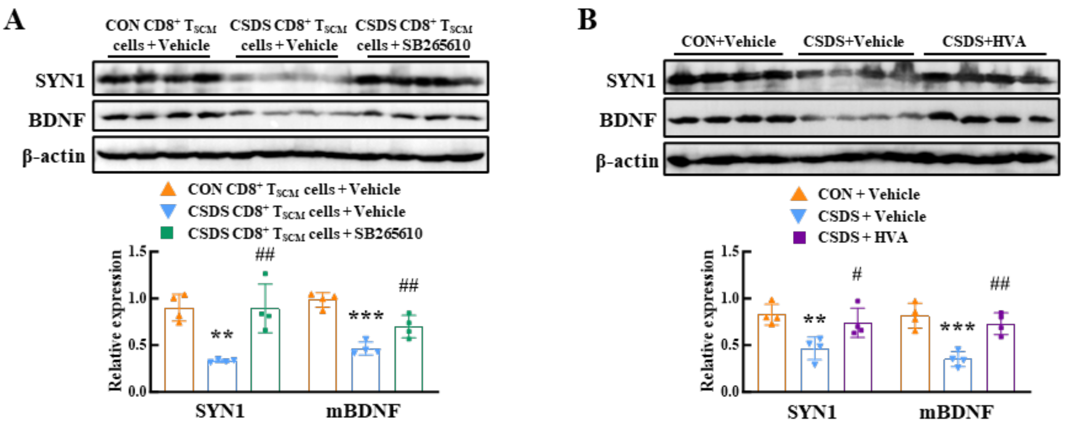

Supplementary Fig. 15 The expression of SYN1 and BDNF. (A) Representative western blots showing the levels of SYN1 and BDNF in the hippocampus of *Rag1*$^{-/-}$ mice. **P = 0.0034 (SYN1) and ***P < 0.0001 (BDNF) versus the control CD8$^+$ T$_{SCM}$ cells group, $^{##}P$ = 0.0034 (SYN1) and $^{##}P$ = 0.0056 (BDNF) versus the CSDS CD8$^+$ T$_{SCM}$ cells group using one-way ANOVA followed by two-sided Holm-Sidak post hoc multiple comparison test, n = 4/group. **(B)** Representative western blots showing the levels of SYN1 and BDNF in the hippocampus of CSDS mice. **P = 0.0092 (SYN1) and ***P = 0.0007 (BDNF) versus the CON+Vehicle group, $^{#}P$ = 0.038 (SYN1) and $^{##}P$ = 0.002 (BDNF) versus the CSDS+Vehicle group using one-way ANOVA followed by two-sided Holm-Sidak post hoc multiple comparison test, n = 4/group. Data are presented as mean±SD. Source data are provided as a Supplementary Source Data file. CON: control, CSDS: chronic social defeat stress, CD8$^+$ T$_{SCM}$ cells: stem cell-like memory CD8$^+$ T cells, HVA: homovanillic acid.

Supplementary Fig. 16

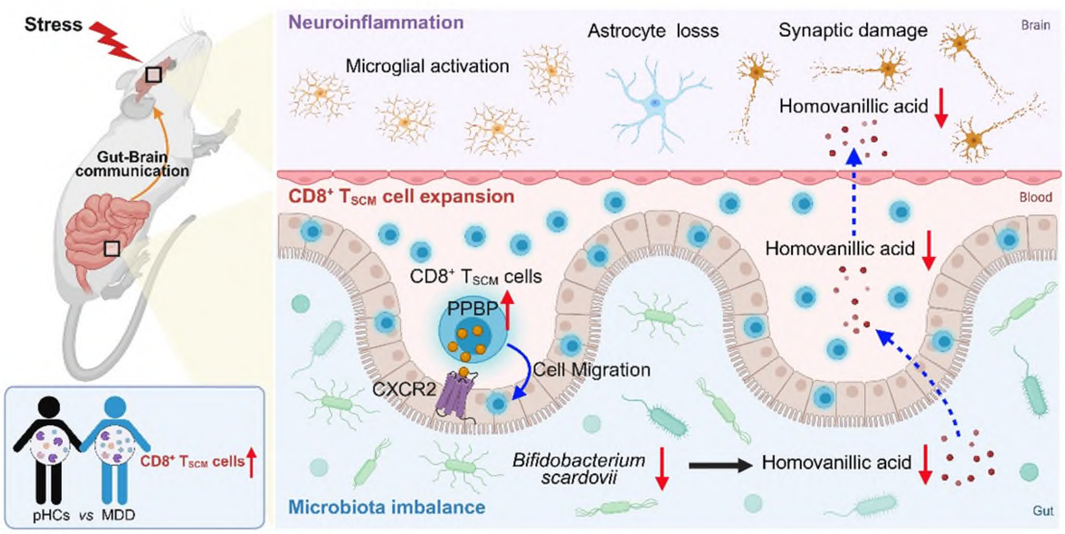

Supplementary Fig. 16 Graphical abstract. We observed a significant enrichment of the CD8$^+$ T$_{SCM}$ cell subset in patients with MDD, which positively correlating with the severity of depressive symptoms. Mechanistically, pathological CD8$^+$ T$_{SCM}$ cells migrated to the intestine in a PPBP-CXCR2 axis-dependent manner. This infiltration induced intestinal inflammation and was followed by a marked reduction in *Bifidobacterium scardovii*, leading to decreased production of homovanillic acid. This metabolic perturbation triggers neuroinflammation and synaptic damage, ultimately contributed to depressive behaviors. Created in BioRender. Yao, H. (2025) https://BioRender.com/pmry4wv. pHCs: psychiatrically healthy controls, MDD: major depressive disorder, CD8$^+$ T$_{SCM}$ cells: stem cell-like memory CD8$^+$ T cells.
