# Supplementary material for: Migration of CD8 + TSCM cells into intestine via PPBP–CXCR2 axis increases host stress susceptibility by inhibiting gut microbiome-derived homovanillic acid
Source: Nat Commun. 2025 Nov 19;16:10165. doi: 10.1038/s41467-025-65112-4 (PMC12630981; doi:10.1038/s41467-025-65112-4)
Supplement: Supplementary file 2 — Description of Additional Supplementary Files [file 41467_2025_65112_MOESM2_ESM.pdf]

Supplementary Data

This supplementary file included Supplementary Data 1-18 as Excel files, which contain the clinical characteristics of psychiatrically healthy controls and MDD patients, antibodies used for flow cytometry, correlation analyses results, and analysis data from the single-cell RNA sequencing and transcriptome sequencing.

Supplementary Movie 1

The 3D distribution of $CD8^+$ T_{SCM} cells (green) in the vDISCO-cleared brain of the *Rag1*$^{-/-}$ recipient mice, related to Figure 4D. The EGFP-labeled $CD8^+$ T_{SCM} cells isolated from control mice, which were then adoptively transferred into *Rag1*$^{-/-}$ mice.

Supplementary Movie 2

The 3D distribution of $CD8^+$ T_{SCM} cells (green) in the vDISCO-cleared brain of the *Rag1*$^{-/-}$ recipient mice, related to Figure 4D. The EGFP-labeled $CD8^+$ T_{SCM} cells isolated from CSDS mice, which were then adoptively transferred into *Rag1*$^{-/-}$ mice.

Supplementary Movie 3

The 3D distribution of $CD8^+$ T_{SCM} cells (green) in the vDISCO-cleared intestine of the *Rag1*$^{-/-}$ recipient mice, related to Figure 4E. The EGFP-labeled $CD8^+$ T_{SCM} cells isolated from control mice, which were then adoptively transferred into *Rag1*$^{-/-}$ mice.

Supplementary Movie 4

The 3D distribution of $CD8^+$ T_{SCM} cells (green) in the vDISCO-cleared intestine of the *Rag1*$^{-/-}$ recipient mice, related to Figure 4E. The EGFP-labeled $CD8^+$ T_{SCM} cells isolated from CSDS mice, which were then adoptively transferred into *Rag1*$^{-/-}$ mice.
