# Supplementary material for: Migration of CD8 + TSCM cells into intestine via PPBP–CXCR2 axis increases host stress susceptibility by inhibiting gut microbiome-derived homovanillic acid
Source: Nat Commun. 2025 Nov 19;16:10165. doi: 10.1038/s41467-025-65112-4 (PMC12630981; doi:10.1038/s41467-025-65112-4)
Supplement: Supplementary file 8 — Reporting Summary [file 41467_2025_65112_MOESM8_ESM.pdf]

Corresponding author(s): Bing Han, Yonggui Yuan, Honghong Yao

Last updated by author(s): Jul 24, 2025

Reporting Summary

Nature Portfolio wishes to improve the reproducibility of the work that we publish. This form provides structure for consistency and transparency in reporting. For further information on Nature Portfolio policies, see our Editorial Policies and the Editorial Policy Checklist.

Statistics

For all statistical analyses, confirm that the following items are present in the figure legend, table legend, main text, or Methods section.

n/a	Confirmed	
☐	☒	The exact sample size (*n*) for each experimental group/condition, given as a discrete number and unit of measurement
☐	☒	A statement on whether measurements were taken from distinct samples or whether the same sample was measured repeatedly
☐	☒	The statistical test(s) used AND whether they are one- or two-sided<br>*Only common tests should be described solely by name; describe more complex techniques in the Methods section.*
☒	☐	A description of all covariates tested
☐	☒	A description of any assumptions or corrections, such as tests of normality and adjustment for multiple comparisons
☐	☒	A full description of the statistical parameters including central tendency (e.g. means) or other basic estimates (e.g. regression coefficient) AND variation (e.g. standard deviation) or associated estimates of uncertainty (e.g. confidence intervals)
☐	☒	For null hypothesis testing, the test statistic (e.g. *F*, *t*, *r*) with confidence intervals, effect sizes, degrees of freedom and *P* value noted<br>*Give P values as exact values whenever suitable.*
☒	☐	For Bayesian analysis, information on the choice of priors and Markov chain Monte Carlo settings
☒	☐	For hierarchical and complex designs, identification of the appropriate level for tests and full reporting of outcomes
☐	☒	Estimates of effect sizes (e.g. Cohen's *d*, Pearson's *r*), indicating how they were calculated

Our web collection on statistics for biologists contains articles on many of the points above.

Software and code

Policy information about availability of computer code

Data collection	We used the following software for data collection: Cell Ranger (v3.1.0)
Data analysis	We used the following software for data analysis: R (v3.6.3) package Seurat (v3.1.1), SingleR (v1.0.1), Metascape (www.metascape.org), CellPhoneDB (https://www.CellPhoneBD.org/), MetaboAnalyst 6.0, ggplot2 (3.4.0), pheatmap (1.0.12),OmicStudio tools, Neurolucida 360, NeuroExplorer, Image J, Hisat2 (2.0.4), Cytoscape software (v3.4.0), MCODE plugin (v1.4.2), FlowJo software 10.6. GraphPad Prism 8.0.1.

For manuscripts utilizing custom algorithms or software that are central to the research but not yet described in published literature, software must be made available to editors and reviewers. We strongly encourage code deposition in a community repository (e.g. GitHub). See the Nature Portfolio guidelines for submitting code & software for further information.

Data

Policy information about availability of data

All manuscripts must include a data availability statement. This statement should provide the following information, where applicable:

- Accession codes, unique identifiers, or web links for publicly available datasets
- A description of any restrictions on data availability
- For clinical datasets or third party data, please ensure that the statement adheres to our policy

The scRNA-seq data generated in this study have been deposited in the National Genomics Data Center database under accession code PRJCA009116 (HRA002269) [https://ngdc.cncb.ac.cn/search/specific?db=hra&q=PRJCA009116]. The metagenomics data generated in this study have been deposited in the National Genomics Data Center database under accession code PRJCA029686 (HRA008457, human microbiota) [https://ngdc.cncb.ac.cn/search/specific?db=hra&q=PRJCA029686] and

PRJCA029610 (mouse microbiota) [https://ngdc.cncb.ac.cn/search/specific?db=bioproject&q=PRJCA029610]. The metabolomics data used in this study are available in the National Genomics Data Center database under accession code PRJCA029658 (mouse brain tissue) [https://ngdc.cncb.ac.cn/search/specific?db=bioproject&q=PRJCA029658] and PRJCA029659 (mouse microbiota) [https://ngdc.cncb.ac.cn/search/specific?db=bioproject&q=PRJCA029659]. The raw data of RNA-seq are accessible on GEO using the accession number GSE200957 [https://www.ncbi.nlm.nih.gov/geo/query/acc.cgi?acc=GSE200957]. All the data supporting this study are available within the article, the Supplementary file, and the Source data file, as indicated in the Reporting summary for this article. Source data are provided with this paper.

Research involving human participants, their data, or biological material

Policy information about studies with human participants or human data. See also policy information about sex, gender (identity/presentation), and sexual orientation and race, ethnicity and racism.

Reporting on sex and gender	We collected samples from 63 men and 167 women, and the sex was not considered in study design.
Reporting on race, ethnicity, or other socially relevant groupings	*Please specify the socially constructed or socially relevant categorization variable(s) used in your manuscript and explain why they were used. Please note that such variables should not be used as proxies for other socially constructed/relevant variables (for example, race or ethnicity should not be used as a proxy for socioeconomic status).* *Provide clear definitions of the relevant terms used, how they were provided (by the participants/respondents, the researchers, or third parties), and the method(s) used to classify people into the different categories (e.g. self-report, census or administrative data, social media data, etc.)* *Please provide details about how you controlled for confounding variables in your analyses.*
Population characteristics	The 230 subjects included 115 major depressive disorder (MDD) patients and 115 healthy controls. All available demographic characteristics for MDD patients and healthy controls are listed in Supplementary Table 1, 2, 5, 9 and 17.
Recruitment	MDD patients were recruited from ZhongDa Hospital, Southeast University, the Third People's Hospital of Huzhou, and the Third People's Hospital of Huai'an, and healthy controls were recruited through socially-oriented advertising in Nanjing and Huai'an from January to June 2021. Diagnoses of neuropsychiatric pathology were conducted by experienced psychiatrists and were based on the Diagnostic and Statistical Manual of Mental Disorders, 5th Edition (DSM-5)
Ethics oversight	The study procedures were approved by The Clinical Research Ethics Committee of ZhongDa Hospital, Southeast University (ID:2020ZDSYLL247-P01).

Note that full information on the approval of the study protocol must also be provided in the manuscript.

Field-specific reporting

Please select the one below that is the best fit for your research. If you are not sure, read the appropriate sections before making your selection.

- [x] Life sciences
- [ ] Behavioural & social sciences
- [ ] Ecological, evolutionary & environmental sciences

For a reference copy of the document with all sections, see nature.com/documents/nr-reporting-summary-flat.pdf

Life sciences study design

All studies must disclose on these points even when the disclosure is negative.

Sample size	Sample size for each experiment is stated in the figure legends. Sample size was not predetermined by statistical methods, but chosen based on previous experience with similar experiments. Data were collected from two or more biological replicates.
Data exclusions	For scRNA-seq study, low-quality cells meeting one of the following thresholds were further excluded: 1) the number of expressed genes was lower than 500 or larger than 4,000; 2) the UMI counts were lower than 2,000 or larger than 15,000 per cell; 3) more than 8% of UMIs were mapped to mitochondrial or ribosomal genes.
Replication	All experiments were repeated and reliably reproduced. Number of mice used and independent experiments performed are indicated in the figure legends.
Randomization	Subjects were assigned to groups based on diagnosis and not by random assignment. All groups were matched for age and gender. For transplantation experiments animals were selected randomly from the same cage for different treatments.
Blinding	Clustering of single nuclei was performed in an unbiased blinded manner. Cluster names were assigned after generation of clusters. Clinicians were blinded for final psychiatry autopsy diagnosis of MDD case or control. Behavioral tests were performed by an investigator blinded to the experimental groups. Gating strategies for flow cytometry analysis were kept the same for control and experimental groups. For quantifications of histological sections and WB, blinding was performed by labeling the sections numerically without prior knowledge of the treatment of the sample.

Reporting for specific materials, systems and methods

We require information from authors about some types of materials, experimental systems and methods used in many studies. Here, indicate whether each material, system or method listed is relevant to your study. If you are not sure if a list item applies to your research, read the appropriate section before selecting a response.

Materials & experimental systems

n/a	Involved in the study
☐	☒ Antibodies
☒	☐ Eukaryotic cell lines
☒	☐ Palaeontology and archaeology
☐	☒ Animals and other organisms
☒	☐ Clinical data
☒	☐ Dual use research of concern
☒	☐ Plants

Methods

n/a	Involved in the study
☒	☐ ChIP-seq
☐	☒ Flow cytometry
☒	☐ MRI-based neuroimaging

Antibodies

Antibodies used

IF:
anti-GFAP antibody (1:400, G3893, Sigma-Aldrich, USA), anti-Iba-1 antibody (1:250, 019-19741, Wako Pure Chemicals, Japan), Alexa 488-conjugated goat anti-mouse IgG (1:300, A11001, Invitrogen), anti-rabbit IgG (1:300, A32731, Invitrogen, USA), CD8a Monoclonal Antibody (1:50, 42-0081-82, Thermo fisher, USA).

WB:
ZO-1 (1:1000, 21773-1-AP, Proteintech Group, China), Occludin (1:1000, 27260-1-AP, Proteintech Group, China), Claudin-5 (1:1000, AF5216, Affinity, USA), SYN1 (1:500, 20258-1-AP, Proteintech Group, China), BDNF (1:500, 28205-1-AP, Proteintech Group, China), GADPH (1:3000, 60004-1-AP, Proteintech Group, China) and β-actin (1:2000, 60008-1-AP, Proteintech Group, China), horseradish peroxidase-conjugated goat anti-mouse/rabbit IgG secondary antibody (1:2000, 7076P2/7074P2, Cell Signaling, USA)

Flow Cytometry:
Fixable Viability Stain 780 (BD Pharmingen, 565388), Alexa Fluor® 700 anti-human CD45 (Biolegend, 304024) PerCP anti-human CD3 (Biolegend, 317338), Brilliant Violet 785™ anti-human CD56 (NCAM) (Biolegend, 362550), Brilliant Violet 421™ anti-human HLA-DR (Biolegend, 307636), Brilliant Violet 650™ anti-human CD19 (Biolegend, 302238), PerCP anti-human CD3 (Biolegend , 300326),BV711 Mouse anti-Human CD56 (BD Pharmingen, 742661), Brilliant Violet 785 anti-human CD4 (Biolegend, 300553), PE-Texas Red CD8 Monoclonal Antibody (3B5) (ThermoFisher , MHCD0817). FITC anti-human CD45RA (Biolegend, 304106), Brilliant Violet 650™ anti-human CD197 (CCR7) (Biolegend, 353234), Brilliant Violet 605 anti-human CD2[illegible] , 302632),PE anti-human CD127 (IL-7Rα) (Biolegend, 351304), Brilliant Violet 510 anti-human CD95 (Fas) (Biolegend , 305640).

Validation

Antibodies were validated by the manufacturer and if produced in house they were validated and optimized by titration prior to use.

Animals and other research organisms

Policy information about studies involving animals; ARRIVE guidelines recommended for reporting animal research, and Sex and Gender in Research

Laboratory animals

Adult male C57BL/6J mice and Rag1-/- mice (25.0-30.0 g, 6-8 weeks old) on a C57BL/6J background

Wild animals

No wild animals were used.

Reporting on sex

All animals were male on this study.

Field-collected samples

No field-collected samples were used.

Ethics oversight

The care and use of animals were reviewed and approved by the Institutional Animal Care and Use Committee at the Medical School of Southeast University (approval ID: 20190222004).

Note that full information on the approval of the study protocol must also be provided in the manuscript.

Plants

Seed stocks

Report on the source of all seed stocks or other plant material used. If applicable, state the seed stock centre and catalogue number. If plant specimens were collected from the field, describe the collection location, date and sampling procedures.

Novel plant genotypes

Describe the methods by which all novel plant genotypes were produced. This includes those generated by transgenic approaches, gene editing, chemical/radiation-based mutagenesis and hybridization. For transgenic lines, describe the transformation method, the number of independent lines analyzed and the generation upon which experiments were performed. For gene-edited lines, describe the editor used, the endogenous sequence targeted for editing, the targeting guide RNA sequence (if applicable) and how the editor was applied.

Authentication

Describe any authentication procedures for each seed stock used or novel genotype generated. Describe any experiments used to assess the effect of a mutation and, where applicable, how potential secondary effects (e.g. second site T-DNA insertions, mosiacism, off-target gene editing) were examined.

Flow Cytometry

Plots

Confirm that:

- [x] The axis labels state the marker and fluorochrome used (e.g. CD4-FITC).
- [x] The axis scales are clearly visible. Include numbers along axes only for bottom left plot of group (a 'group' is an analysis of identical markers).
- [x] All plots are contour plots with outliers or pseudocolor plots.
- [x] A numerical value for number of cells or percentage (with statistics) is provided.

Methodology

Sample preparation	Peripheral blood mononuclear cells (PBMCs) were obtained from whole blood samples by density gradient centrifugation. After the plasma was collected by centrifugation, Ficoll-Paque Plus (17144002, cytiva, Sweden) was placed at the bottom of the centrifuge tube, the whole blood was diluted to 1 × PBS in a 1:1 ratio and then tiled on the top of Ficoll-Paque Plus, centrifuged at 500 g for 30 minutes. The centrifugal PBMC layer was then collected, and incubated with ACK lysis buffer (C3702, Beyotime Biotech, China) for 5 minutes at 4℃ to remove red blood cells. PBMCs were collected by centrifugation and then re-suspended with 1 ml 1 × PBS. Finally, cell suspensions were filtered by a 70-µm cell strainer. Cell viability > 90% was required for subsequent construction of libraries.
Instrument	BD FACSCelestaTM Flow Cytometer
Software	FlowJo software 10.6 was used to analyse data
Cell population abundance	*Describe the abundance of the relevant cell populations within post-sort fractions, providing details on the purity of the samples and how it was determined.*
Gating strategy	To investigate the proportion of cell immune subsets in PBMCs of human, PBMCs samples were gated for T cells (CD45+CD3+), Myeloid cells (CD45+CD3-CD19-HLA-DR+), NK cells (CD45+CD3-CD56+), B cells (CD45+CD3-CD19+), regulatory CD4+ T (CD4+ Treg) cells (CD45+CD3+CD56-CD4+CD25+CD127low), CD4+ Naïve T cells (TNaïve) cells (CD45+CD3+CD56-CD4+CD25-CD45RA+CCR7+), CD4+ central memory T (TCM) cells (CD45+CD3+CD56-CD4+CD25-CD45RA-CCR7+), CD4+ effector memory T (TEM) cells (CD45+CD3+CD56-CD4+CD25-CD45RA-CCR7-), CD8+ TNaïve cells (CD45+CD3+CD56-CD8+CD45RA+CCR7+CD95-), stem cell-like memory CD8+ T (CD8+ TSCM) cells (CD45+CD3+CD56-CD8+CD45RA+CCR7+CD95+), CD8+ TCM cells (CD45+CD3+ CD56-CD8+CD45RA-CCR7+), CD8+ TEM cells (CD45+CD3+CD56-CD8+CD45RA-CCR7-), and terminally differentiated effector memory CD8+ T (CD8+ TEMRA) cells (CD45+CD3+CD56-CD8+CD45RA+CCR7-).<br><br>To investigate the proportion of CD8+T cell subsets in PBMCs of mice, samples were gated for CD8+ TNaïve cells (CD45+CD3+NK1.1-CD8+CD62L+CD44-Sca-1-), CD8+ TSCM cells (CD45+CD3+NK1.1-CD8+CD62L+CD44-Sca-1+), CD8+ TCM cells (CD45+CD3+NK1.1-CD8+CD62L+CD44+), CD8+ TEM cells (CD45+CD3+NK1.1-CD8+CD62L-CD44+), and tissue-resident memory CD8+ T cells (CD8+ TRM) cells (CD45+CD3+NK1.1-CD8+CD62L-CD44-).

- [x] Tick this box to confirm that a figure exemplifying the gating strategy is provided in the Supplementary Information.
